# Healthcare middle managers’ experiences of developing capacity and capability: a systematic review and meta-synthesis

**DOI:** 10.1186/s12913-019-4345-1

**Published:** 2019-08-05

**Authors:** Trude Anita Hartviksen, Jessica Aspfors, Lisbeth Uhrenfeldt

**Affiliations:** 1 Faculty of Nursing and Health Sciences, Nord University, Bodø, Norway; 2 Faculty of Education and Arts, Nord University, Bodø, Norway; 3 Faculty of Nursing and Health Sciences, Nord University, Bodø, Norway

**Keywords:** Healthcare middle manager, Leadership, Complexity, Capacity, Capability, Development, Empowerment, Systematic review, Meta-synthesis

## Abstract

**Background:**

Healthcare middle managers play a central role in reducing harm, improving patient safety, and strengthening the quality of healthcare. The aim of this systematic review was to identify the present knowledge and critically discuss how healthcare middle managers experienced to develop the capacity and capability for leadership in a healthcare system characterized by high complexity.

**Methods:**

This comprehensive systematic review provided evidence of healthcare middle managers’ experiences in developing the capacity and capability for leadership in public healthcare. The three-step literature search was based on six databases and led by a PICo question. The review had a critical hermeneutic perspective and was based on an a priori published, protocol. The methods were inspired by the Joanna Briggs Institute and techniques from Kvale and Brinkmann. The results were illustrated by effect size, inspired by Sandelowski and Barroso.

**Results:**

Twenty-three studies from four continents and multiple contexts (hospitals and municipal healthcare) published from January 2005–February 2019 were included. Based on experiences from 482 healthcare middle managers, 2 main themes, each with 2 subthemes, were identified, and from these, a meta-synthesis was developed: *Healthcare middle managers develop capacity and capability through personal development processes empowered by context.* The main themes included the following: 1. personal development of capacity and capability and 2. a need for contextual support. From a critical hermeneutic perspective, contrasts were revealed between how healthcare middle managers experienced the development of their capacity and capability and what they experienced as their typical work situation.

**Conclusions:**

This review provides evidence of the need for a changed approach in healthcare in relation to criticisms of present organizational structures and management methods and suggestions for how to strengthen healthcare middle managers’ capacity and capability for leadership in a healthcare system characterized by high complexity. Evidence of how leadership development affected the clinical context and, thus, the quality of healthcare was found to be a field requiring further research.

**PROSPERO registration number:**

CRD42018084670

## Background

Healthcare middle managers (HMMs) were recognized in this systematic review as the leadership level closest to everyday clinical practice [1, 2], any manager who is supervised by an organization’s top manager and who supervise one level above line workers and professionals [2, 3]. This leadership level is often referred to as first or frontline leaders, nursing leaders, or clinical managers. This review included HMMs in public healthcare services. HMMs have extensive responsibility in healthcare organizations [1]. Their central position, between executives and frontline employees, makes HMMs crucial in limiting knowledge and information gaps [4, 5] and translating top-level policies, strategies and means to improve patient quality and reduce harm [6]. Positive leadership has been related to increased patient satisfaction, fewer adverse events [7, 8], lower patient mortality, medication errors and restraint use, and fewer hospital-acquired infections [8]. Nursing leadership directly and indirectly influences nurses’ motivations [9]. Close to the organizational context, HMMs possess unique knowledge, skills and experience [3], depending on their individual and the organization’s capacity and capability. Capacity includes individual features such as technical expertise, creative thinking skills, social skills, and organizational understanding. Capability includes what HMMs are able to implement, such as the ability to identify and define problems and handle complex contexts [10], the ability to adapt to change, generate new knowledge and continuously improve [11].

HMMs’ capacity and capability have been shown to develop through several different individual and collaborative approaches. These approaches have included learning specific competencies through cognitive, social, and technical strategies, system thinking, personal mastery, mental models, the development of a shared vision, team learning, training, programmes, management systems and coaching [12]. Developing assignments, feedback and training in actual organizational challenges, and the prioritization of leadership development in the organization have proven to be good strategies [13, 14]. HMMs’ development of capacity and capability involves self-awareness [14] and changing integrated cultures, attitudes and habits [12]. However, leadership development programs have had a tendency to focus on skills training and technical and conceptual knowledge, and to a lesser extent on personal growth and awareness [15].

Leadership development consists of multilevel and longitudinal dynamic complex processes [11, 14]. It has been suggested that the job satisfaction of HMMs improves through the decentralization of the organizational structure, increased organizational support from supervisors and through empowering HMMs to participate in decision making [16]. Interventions based on actions, audits, feedback, reminders and various types of education have proven to be more effective in changing professional behaviour than persuasion-based actions, such as local consensus processes and opinion leaders [17]. Quality improvement collaboratives have been widely used as an approach to shared learning and improvement in healthcare and have been shown to improve targeted clinical processes and patient outcomes [18]. Findings related to educational development and job training have been inconclusive and require further research [16]. It is claimed that the development of leadership in healthcare organizations requires a cooperative approach that achieves the best results when it incorporates the local context [19].

Healthcare is a context of increasing complexity that is generally acknowledged to be complex social systems [20]. This increasing complexity refers to a rapidly changing healthcare system with new technology and treatment methods and increasing focus on coherent, proactive person-centred services, a context that alters the prerequisites for HMMs’ capacity and capability [21, 22]. The nonlinear, dynamic, and unpredictable nature of healthcare [20–24] has been described through various perspectives of system theory and complexity theory; complex adaptive systems (CAS) and complex responsive processes (CRP) are examples of these perspectives [21]. CAS describes how individual agents in healthcare systems are free to act in unpredictable and interconnected ways [25]. Stacey et al. [26] introduced CRP, which attempts to understand human organizations as processes. This approach was seen as new and necessary in order to differentiate and distance itself from the dominating understanding of human organizations as objectifying systems and rationalistic causality. CRP emphasizes human interaction as the basis of transformative organizations. The difference between CAS and CRP could be described as the difference between a mathematical (CAS) or social (CRP) perspective on complexity. The perspectives could also be combined into a contextual complexity perspective, allowing the possibility of contextually shifting between perspectives [21].

Complex systems are based on collective behaviours in dynamic networks, where continuous changes are necessary and occur regularly [27]. In this context, HMMs have experienced a shift from professional authority to managerial values, economic stress [9], dominating top-down management and a loss of involvement and autonomy. These changes have been associated with multiple reforms beginning in the 1980s that aimed to manage public service organizations using private sector principles; these reforms are known as the New Public Management approach [3, 28]. Rather than adapting the leadership style to the tasks at hand, the staff and their previous experiences, leaders tend to favour a preferred leadership style, predominantly transactional leadership [20]. It has, however, been shown to be difficult to achieve changes through command and control strategies [27]. It has been argued that a dynamic, emerging, creative and intuitive view of healthcare should replace the traditional “reduce and resolve” perspective [25]. This approach involves developing new principles in healthcare leadership [21–24], accepting that some behaviours emerge self-organized, and accepting that minimum specifications [28], aims, limits and incentives [29] are better approaches than long-range plans and targets [28].

The expedient choice of leadership style is known to be situational. Given this understanding, the complexity in healthcare organizations requires leadership development that provides the capability to modify leadership styles [14]. Diverse leadership styles have been found to be positively associated with nurse, patient and organizational outcomes [30]. It has been suggested that healthcare needs to encourage and develop transformational [20, 31], collaborative, reflective [20] and relational leadership styles [20, 31, 32], such as authentic leadership [33]. Transformational leadership has been shown to improve patient outcomes [6], increase well-being and decrease burnout factors in staff [34]. Relational leadership has been shown to increase job satisfaction [32, 33], patient satisfaction [7], retention, work environment factors, individual production [32], structural empowerment, work engagement and trust and to decrease negative workplace behaviours and burnout [33], adverse events, medication errors, restraint use, hospital-acquired infections and patient mortality [8].

HMMs’ development of the capacity and capability for leadership in the present complex healthcare context is a field in need of more knowledge [14, 35–38]. The aim of this systematic review was to identify the existing knowledge in this field and to critically discuss how HMMs experienced to develop the capacity and capability for leadership in a healthcare system characterized by high complexity.

## Methods

The methodological perspective in this systematic review was a critical hermeneutic perspective [39, 40]. The critical perspective indicates that this review not only aimed to produce evidence but also to elucidate when theoretical statements represented changeable dependent relationships, which is often taken for granted. This approach involved looking for contrasts to what HMMs experienced developed their capacity and capability for leadership in relation to HMMs’ life world and system world [41]. The critical perspective was supported by a critical appraisal process in which the first and third reviewer cooperated closely, and the second reviewer was available in cases of disagreement. The overall hermeneutic perspective denoted that knowledge was interpreted through the interpreters’ preunderstanding, where the comprehension of the whole affected the understanding of the parts, and the interpretation of the parts was based on the comprehension of the whole [39].

All three reviewers were experienced in knowledge development. The first and third reviewers had practical experience with capacity and capability development in complex healthcare contexts and performing and researching healthcare leadership with a critical perspective [42–44]. The second reviewer was experienced in capacity building, research on teachers’ professional development [45], and research on healthcare leadership with a critical perspective [44].

This comprehensive systematic review was based on an a priori published, peer-reviewed protocol [12], which implies similarities in the design and methods between this review and the published protocol. Both the review and protocol were inspired by the meta-aggregation guidelines established in the Joanna Briggs Institute (JBI) Reviewers’ Manual for qualitative studies [46–48], where both the appraisal and extraction processes before the synthesis added to the critical perspective. The aggregation combined the parts into a whole that was more than the sum of the individual results, which is analogous to a meta-analysis. Based on the a priori published, peer-reviewed protocol [12], the method involved a process of seven steps: 1. formulating a PICo question (Participants, phenomena of Interest, Context), 2. developing a search strategy, 3. searching for knowledge, 4. selecting studies, 5. critically appraising studies, 6. extracting and analysing data and 7. synthesizing data [46]. These seven steps were implemented while conducting this review and were followed up through the presentation of the methods and results. To increase the trustworthiness of the results, in step 6, we calculated the effect size for each theme based on the number of studies providing evidence for each theme. The choice of calculating effect size was based on Sandelowski [49], who described how using numbers provides a better illustration of patterns, sharpens the focus, and adds to the validity by verifying analytical moves.

### Search strategy

The three-step search strategy followed the a priori published, peer-reviewed protocol [12]. The search strategy was based on the following PICo question [46]: The participants (P) were HMMs, as the leaders closest to public healthcare practice, with responsibility for both clinical practice and healthcare personnel. Studies were included irrespective of how long the HMMs had been in a leadership position and regardless of their professional backgrounds. Studies of HMMs without personnel responsibilities were excluded. The phenomena of interest (I) were studies that described, investigated, or explored how HMMs experienced the development of the capacity and capability for leadership. Thus, the review considered studies that focused on qualitative data. The context (Co) included the complexity in community and specialized healthcare and was limited to public healthcare services. The purpose of this limitation was to consider the contextual meaning of public healthcare as different from non-public healthcare [50]. The PICo question described the focus, scope and applicability of this review [46] and was used to clarify the search, as demonstrated in Table 1.

**Table 1:** PICo question and search terms

	Participants: Healthcare middle managers	*Boolean operator:*	Interest of phenomena: Developing the capacity and capability for leadership	*Boolean operator:*	Context:Complexity in public healthcare services
Search terms, step 1	Middle manager OR First-line manager OR Leadership ORLeaders	*AND*	Developing OR Learning OR Capacity OR Capability OR	*AND*	Healthcare OR Complexity
Final search terms, step 2	Leaders* OR Nurse leaders* OR Nurse administrators OR Nurse manage* OR Hospital administrators OR Health facility administrators OR Middle manage* OR Nursing manage* OR Personnel manage* OR Quality manage*	*AND*	Capacity building OR Capabilities OR Competence OR Development	*AND*	Health care OR Health care system OR Healthcare system OR Public sector OR Health care sector OR Delivery of Health Care OR Delivery of healthcare OR Healthcare delivery OR Health care delivery OR Complexity

*Indicates truncation; cutting the end of the search term to expand the search

The search process started in October 2017 with step 1, which was a preliminary search identifying whether any current or ongoing systematic reviews on this or similar topics existed. No such reviews were identified. Studies published in English, German, Swedish, Norwegian, and Danish between January 2005 and February 2019 were considered for inclusion. The languages were chosen based on the reviewers’ common linguistic platform. The time limitation was chosen due to the rapidly changing complexity of the last decades in industrialized countries’ healthcare, including an increased focus on user involvement, interdisciplinarity, and interdepartmental cooperation [21–25, 34–36, 51–58]. Step 1 expanded the list of relevant search terms. Based on a dominant scope of nursing-related research, such search terms were included in addition to the multidisciplinary search terms. Step 1 revealed HMM to be the most common international multidisciplinary terminology to describe this level of leadership in healthcare.

**Fig. 1:**
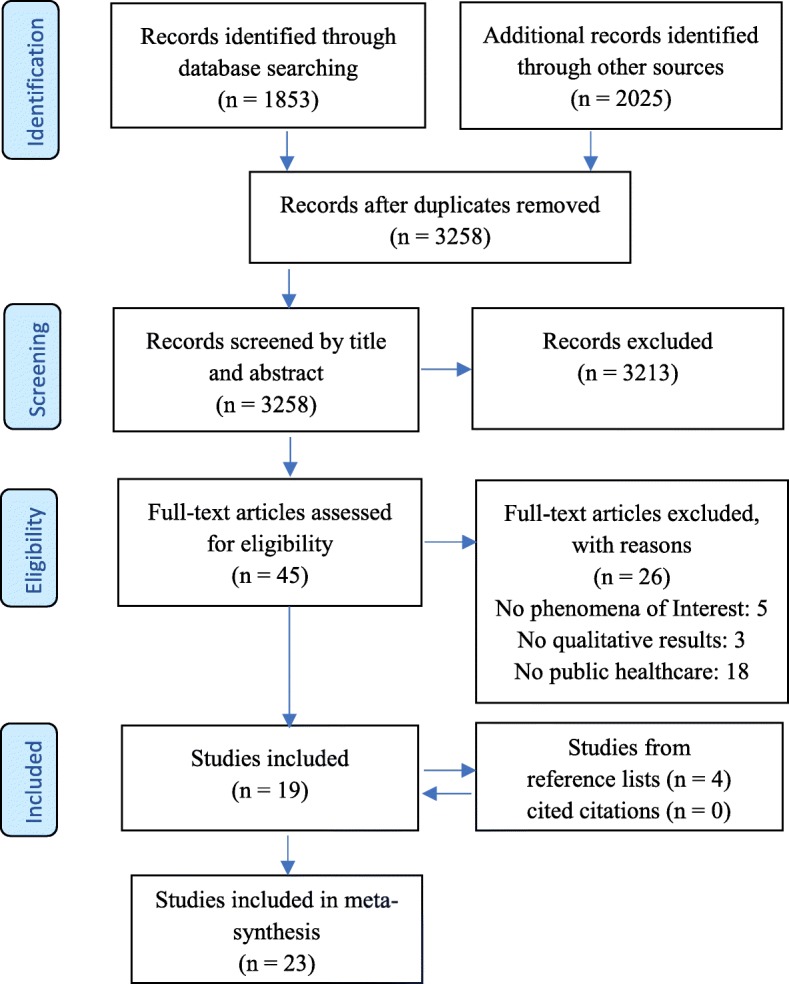
PRISMA 2009 Flow Diagram

In step 2, the comprehensive literature search aimed to find both published and unpublished studies [12]. Based on Sandelowski [49], we added berry-picking. The databases searched were PubMed, CINAHL and Scopus. The search for grey literature included Google Scholar, MedNar and ProQuest Dissertations and Theses Global. The searches were performed in cooperation with two university librarians from Nord University. MeSH terms (Medical Subject Headings) or headings were used when possible. The identified studies were referenced using EndNote as a selection tool. In step 3, the reference lists of the initially included studies and studies that cited the included studies were searched [49, 59]. The process of identifying relevant studies was illustrated in a PRISMA diagram (see Fig. 1). Table 3 summarizes the selected studies.

### Critical appraisal

The retrieved qualitative studies were assessed by two independent reviewers (reviewers 1 and 3) using the standardized ten-item critical appraisal checklist from the JBI: The Qualitative Assessment and Review Instrument (JBI-QARI). A four-point scale (yes, no, unclear, and not applicable) was applied [46]. For questions 1–5, the retrieved studies were assessed for congruity among their stated philosophical perspective, research methodology, research objectives, data collection methods, representation and analysis of data, and the interpretation of their results. For questions 6–10, the studies were assessed to culturally or theoretically locate the researcher and to address the researcher’s influence in order to obtain an adequate representation of participants, ethical issues, and whether the conclusions flowed from the interpretation of data. There were few differences between the reviewers. Those differences that arose were caused by differences in reading the descriptions in the primary studies of the methodology and methods and were resolved through discussions. Table 2 presents the results and percentage achievement from the critical appraisal.

**Table 2:** Results from the critical appraisal of methodological quality (JBI-QARI) [46]

Results from critical appraisal of 23 studies										
Study no/ Question no	1	2	3	4	5	6	7	8	9	10
1. Bergin [60]	Yes	Yes	Yes	Yes	Yes	Yes	Yes	*Unclear*	Yes	Yes
2. Chuang et al. [61]	Yes	Yes	Yes	Yes	Yes	Yes	*Unclear*	*Unclear*	Yes	Yes
3. Clarke et al. [62]	No	*Unclear*	*Unclear*	Yes	*Unclear*	Yes	No	Yes	Yes	Yes
4. Cummings et al. [63]	No	Yes	Yes	*Unclear*	*Unclear*	Yes	No	*Unclear*	No	Yes
5. Debono et al. [64]	Yes	Yes	Yes	Yes	Yes	Yes	No	Yes	Yes	Yes
6. Dellve & Wikstrom [64]	Yes	Yes	Yes	Yes	Yes	Yes	No	*Unclear*	No	Yes
7. Dellve & Eriksson [65]	Yes	Yes	Yes	Yes	Yes	Yes	*Unclear*	*Unclear*	Yes	*Unclea*r
8. Eide et al. [66]	Yes	Yes	Yes	Yes	Yes	Yes	Yes	Yes	Yes	Yes
9. Goodridge et al. [67]	Yes	Yes	Yes	Yes	Yes	Yes	No	Yes	Yes	Yes
10. Hartviksen et al. [44]	Yes	Yes	Yes	Yes	Yes	Yes	Yes	Yes	Yes	Yes
11. Hodgson [68]	Yes	Yes	Yes	Yes	Yes	Yes	Yes	Yes	Yes	Yes
12. Hyrkäs et al. [69]	Yes	Yes	Yes	*Unclear*	*Unclear*	Yes	No	*Unclear*	No	*Unclear*
13. Korhonen & Lammin-takanen [70]	*Unclear*	*Unclear*	*Unclear*	*Unclear*	*Unclear*	Yes	No	Yes	Yes	Yes
14. Lavoie-Tremblay et al. [71]	*Unclear*	Yes	*Unclear*	*Unclear*	*Unclear*	Yes	No	*Unclear*	*Unclear*	Yes
15. Lunts [72]	Yes	Yes	Yes	Yes	Yes	Yes	No	Yes	No	Yes
16. MacPhee et al. [73]	*Unclear*	*Unclear*	*Unclear*	*Unclear*	*Unclear*	*Unclear*	*Unclear*	*Unclear*	*Unclear*	*Unclear*
17. Miltner et al. [74]	*Unclear*	*Unclear*	*Unclear*	*Unclear*	*Unclear*	Yes	*Unclear*	*Unclear*	No	*Unclear*
18. Paliadelis [75]	Yes	Yes	Yes	Yes	Yes	Yes	Yes	Yes	Yes	Yes
19. Paliadelis et al. [76]	Yes	Yes	Yes	Yes	Yes	Yes	No	Yes	Yes	Yes
20. Simpson [77]	Yes	Yes	Yes	Yes	Yes	Yes	Yes	*Unclear*	Yes	Yes
21. Tistad et al. [78]	*Unclear*	*Unclear*	*Unclear*	*Unclear*	*Unclear*	Yes	No	*Unclear*	*Unclear*	*Unclear*
22. Tyan [79]	Yes	Yes	Yes	Yes	Yes	Yes	Yes	*Unclear*	Yes	Yes
23. Udod & Care [80]	*Unclear*	*Unclear*	Yes	*Unclear*	*Unclear*	Yes	No	*Unclear*	*Unclear*	*Unclear*
In total	65%	74%	74%	65%	61%	96%	30%	43%	61%	74%

### Data extraction

The data from the included studies were extracted to a developed meta-summary scheme, which was inspired by the JBI, the System for the Unified Management, Assessment and Review of Information (JBI-SUMARI) [46], which is illustrated in Table 3. The extracted data included specific details about the studies’ origin, aim, participants, methods, context and the results of the significance to the review question. Only aims and results related to HMMs’ development of capacity and capability for leadership were summarized. Only qualitative results were summarized in the included mixed-method studies (*n* = 3).

**Table 3:** Meta-summary of the included studies

Author, year, country	Aim	Participants (*n* = 482) Method	Data analysis	Context	Capacity and capability are described as (Results):
1. Bergin (2009) Sweden [60]	To elucidate processes involved in the way HMMs face and deal with their work situation	10 HMMs (Nurses and physiotherapist) Individual interviews	Grounded theory (Glaser [83, 84], Glaser et al. [85], and Kvale [86])	District hospitals and municipal long-term care	Experiences of defining their own leadership limits; trusting their own assessments and valuing their own competence and experience; creating space for reflection and learning; generating a managerial identity and integrity, respect for human diversity, and self -respect; establishing authority, autonomy, power, and influence
2. Chuang et al. (2011) USA [61]	To understand organizational and relational factors that influence middle managers’ support for innovation implementation processes	92 HMMs (Nurses and environmental services staff) Individual interviews and focus groups	Thematic analysis (Erzberger [87]; Miles et al. [88])	General hospital	Experiences of development of complex innovations and improved performance based on early and often information, maximized discretion, resource availability, upper management support and a learning culture
3. Clarke et al. (2012) Australia [62]	To evaluate the professional development components of the *New South Wales Health Take the Lead Program*	17 HMMs (Nurses) Qualitative questionnaires, individual telephone interviews, and focus groups	Standard quantitative methodology (no ref)	District and general hospitals	Experiences of feeling valued and empowered in an increasingly complex healthcare, developing a network, focusing on reflection, being a role model. Less administrative, more frontline leadership. Appreciation of the role and nursing as a profession, time management, concentration, better strategic planning, positive future outlook
4. Cummings et al. (2014) Canada [63]	To pilot a 2-day coaching workshop conducted as a leadership development strategy	21 HMMs (Nurses) Workshops and focus groups	Iterative approach (no ref)	Municipal long-term care	Experiences of increased intentions to be a coach and coaching skills dealing with complexity. Communication techniques, technique of leading by example. Building confidence and empowering staff. Promoting feedback processes. Trust and respect between HMMs and staff
5. Debono et al. (2014) Australia [64]	To examine the effect of the *Take the Lead Program* on Nursing Unit Managers’ and Midwife Unit Managers’ job performance, leadership skills and the experiences of their patients	60 HMMs (Nurses and midwifes) Individual telephone interviews	Thematic analysis (Creswell et al. [89])	District and general hospitals	Experiences of a multifaceted educational program meeting complexity which enhanced job performance, leadership skills and confidence. Some improved patient experiences. Lean thinking and communication were experienced as most valuable. Improvement in problem-solving and decision-making skills. Collaborative articulation as a result of networking
6. Dellve and Wikstrom (2009) Sweden [82]	To conceptualize how health care leaders may be supported to influence their psychosocial work environment	39 HMMs (Nurses and physicians) Individual interviews and focus groups	Grounded theory (Glaser [83], Glaser et al. [85] and Charmaz [90])	District and general hospitals and municipal healthcare	Experiences of managing complex workplace stress, socializing in formal and informal leadership strategies, strategic leadership structures and occupational identity. Networking increased dialogue, cooperation and understanding. Reflective dialogue, communication and feedback from top managers, staff and human resources. Strategic mentorship programs and multidisciplinary leader development courses. Theoretical and practical knowledge. Self-reflection. Trust. Teamwork
7. Dellve and Eriksson (2017) Sweden [65]	To describe the theoretical framework, i.e., the theoretical underpinnings and pedagogical principles, for leadership programs that support managers’ evidence-based knowledge of health-promoting psychosocial work conditions as well as their capability to apply, adapt, and craft sustainable managerial work practices	44 HMMs (Professional background not described) Individual interviews and focus groups	Unclear (no ref)	District hospitals and municipal healthcare	Experiences of providing a systematic approach for working with complex issues, knowledge and inspiration, reflective dialog. Broader perspectives and concrete tools. Support, encouragement and inspiration from peer managers. Relational coordination. Top management support. Following up at one’s own workplace
8. Eide et al. (2016) Norway [66]	To develop and investigate the feasibility of a 6-week web-based ethical leadership educational program and learn from participants’ experience	9 HMMs (Nurses) Focus groups	Content analysis (Elo and Kyngäs [91])	Municipal long-term care, homecare and health centres	Experiences of reflection and motivation, counteracting a feeling of loneliness and promoting the execution of change. Ethic projects, situational feedback, mindfulness exercises, I’m ok diary, actualized ethical leadership issues, and improvement proposals
9. Goodridge et al. (2015) Canada [67]	To address changes in leadership practices associated with the implementation of Lean, and how the changed practice contributes to subsequent outcomes	4 HMMs (Professional background not described) Workshop, documentary review and individual interviews	A realist coding framework (no ref)	District and general hospitals and municipal healthcare	Experiences of Lean as complex interventions, aligning aims and objectives, attention and resources to quality improvement and change management, tools, changed attitudes or beliefs about leadership, increased levels of expertise, accountability and commitment, measuring and using data effectively, creating or supporting a learning organization culture. Network. Self-confidence. Empowered by autonomy, information, support, resources and professional development
10. Hartviksen et al. (2018) Norway [44]	To identify and discuss the facilitation of HMMs’ development of capacity and capability for leadership	16 HMMs (Nurses) Focus groups	Critical hermeneutic (Kvale and Brinkmann [81], Alvesson and Sköldberg [92])	General hospital, municipal long-term care and homecare	Experiences of trusted interaction despite organizational and structural frames and knowledgeable understanding of complex context, knowledge, trust, and confidence. Transformative learning, coherence, reflection, discussion, repetition, workshops, knowledge sharing, and short lectures. Network. Flexibility. Leadership plan. Changed approach to leadership
11. Hodgson (2015) Canada [68]	To explore the development of self-efficacy in nursing leaders	7 HMMs (Nurses) Individual interviews	Content analysis (Polit and Beck [93])	District and general hospitals	Experiences of horizontal mentoring and developing self-efficacy in complex healthcare systems. Confidence, knowledge, feedback, validation and communication. Observing others. Experience of choosing to sink or swim. Human resources. Relationships with others. Knowing who to call. Support from peers and superiors. Individual strategies. Reflection, following the rules and/or learning by mistakes
12. Hyrkäs et al. (2005) Finland [69]	To explore how first-line managers see future effects of the clinical supervision intervention 1 year after its termination	12 HMMs (Nurses) Short essays	Thematic analysis (no ref)	District hospital	Experiences of positive long-term effects on leadership, leadership role, interaction and communication skills, the desire for self-development, self-knowledge and coping. A broader perspective on work in a complex context, enhanced use of clinical supervision as a supportive measure. Skills in human resource management
13. Korhonen and Lammin-takanen (2005) Finland [70]	To describe nurse managers’ expectations, attitudes and experiences of web-based learning before and after participation in a web-based course	23 HMMs (Nurses) Diagnostic assignments and individual interviews	Content analysis (Cavanagh [94], Insch et al. [95])	District and general hospitals	Experiences of changed attitudes to web-based learning. Lack of recourses limited the development. Developed information technology skills. Professional development as a nurse manager, developing oneself, management skills, and written communication and interaction skills
14. Lavoie-Tremblay et al. (2014) Canada [71]	To describe managers’ and health care providers’ perceptions of the development of their change capacities with the Transforming Care at the Bedside Program in a university-affiliated health care organization	3 HMMs (Nurses) Focus groups and individual interviews	Guided by the interview questions, using NVivo (Polit and Beck [93], Miles et al. [88], Miles et al. [96])	District hospital	Experiences of understanding the bigger picture, structured process to lead change, learning skills, skills to engage team members, better organize and plan changes, group cohesiveness and belonging, awareness of others, work as a team, new relationships, and to make results visible
15. Lunts (2012) United Kingdom [72]	To explore what middle managers perceived as helping them in the delivery of change in one high-profile integration project	6 HMMs (Professional background not described) Individual interviews	Grounded theory (Corbin and Strauss [97])	Municipal healthcare	Experiences of progress, informal networks. Dedicated time and awareness of complexity, leadership models, help to lead change. Clear steering and vision from senior leaders. Clear structures. Trust and respect. Mental models and strategies for working in complexity. Conceptual models and practical guidance on dealing with change within complexity
16. MacPhee et al. (2011) Canada [73]	To describe nurse leaders’ perspectives of the outcomes of a formal leadership program	27 HMMs (Nurses) Individual telephone interviews	Content analysis (Graneheim and Lundman [98])	District and general hospitals, municipal homecare, mental and public health	Experiences of increased self-confidence, positive changes in leadership styles, the importance of communication, reflection and discussions in complex health environments. Fulfil their leadership roles and responsibilities. Feedback from senior management. Leadership skills. Mentoring. Adding recourses and tools. Project management competencies. Change management. Workshops. Nursing focus. Interprofessional courses
17. Miltner et al. (2015) USA [74]	To describe the identified professional development needs of nurse managers in a metropolitan area in the south-eastern United States	20 HMMs (Nurses) Focus groups	Content analysis (Hsieh and Shannon [99])	District and general hospitals	Experiences of learning as you go and gaining a voice navigating complexity, and to garner support. Internal mentoring programs
18. Paliadelis (2005) Australia [75]	To explore nurse unit managers’ stories about the education and support they receive in their role	20 HMMs (Nurses) Individual interviews	Voice-relational method (Gilligan [100], Mauthner and Doucet [101], Doucet and Mauthner [102])	General hospitals	Experiences of a lack of support, individual seeking of suitable sources of management education, peer group support. To sink or swim
19. Paliadelis et al. (2007) Australia [76]	To explore how nurse unit managers cope, what helps them in their role	20 HMMs (Nurses) Individual interviews	Unclear (No ref)	General hospitals	Experiences of lack of formal support and respect in an increasingly complex role, support within own ranks. Sink or swim
20. Simpson (2006) Canada [77]	To identify the enhancers for informal learning, create and support a culture of learning and innovation	9 managers (Number of HMMs and professional background not described) Field work, individual interviews and focus group	Several, interpretivist (Gubrium and Holstein [103], Miles et al. [88])	District hospital	Experiences of informal learning about people, values and culture, knowledge, attitudes and skills. Collaboration, networking and sharing, passion and purpose, trust. Balancing challenges, opportunities and support, learning and creativity, respect. Connection to the organization, empowerment and freedom, modelling, no blame environment, recognition, support and valuing. Conversations and storytelling
21. Tistad et al. (2016) Sweden [78]	To explore the feasibility and usefulness of a leadership intervention to support managers’ implementation of clinical practice guidelines recommendations, considering the influence of the context	11 HMMs (Professional background not described) Fieldwork, individual interviews and individual telephone interviews	Content analysis (Elo and Kyngäs [91], Graneheim and Lundman [98])	Specialized hospitals	Experiences of the participation of senior and frontline managers. Both understanding and templates are required to recognize and manage complexity. Leadership plan, knowledge and skills. Limited impact on managers’ behaviours or clinical practice. Increasing understanding and awareness of their vital role
22. Tyan (2010) Taiwan [79]	To examine the perspectives of Taiwanese nurse managers who participated in a US home healthcare learning tour regarding the development of home healthcare for the elderly in Taiwan and to describe the views of Taiwanese home healthcare nurse managers on empowerment within the context of home healthcare	5 HMMs (Nurses) Focus groups, self-reflective diaries, individual interviews, fieldwork, and qualitative questionnaires	Content analysis (Hsieh and Shannon [99])	District hospitals	Experiences of professional development from taking an international learning tour. Based on the complexity of patient care. Experiences of being empowered on the individual and interpersonal level, but powerless on the system level
23. Udod and Care (2012) Canada [80]	To explore the stress experiences and coping strategies of nurse managers in an acute care setting in Canada to recruit and retain individuals in nurse managers roles	5 HMMs (Nurses) Individual interviews	Content analysis (no ref)	District hospital	Experiences of less effective coping strategies. A need for infrastructure and support systems. Access to continuous professional development, flexible, respond to rapidly changing complex environment

### Data analysis and meta-aggregation

The included qualitative research results were analysed with meaning condensation, which was inspired by Kvale and Brinkmann [81]. This analysis involved an aggregation and synthesis of the results in a critical process, which was a back and forth movement between the parts and the whole, searching for contrasts [40] in what HMMs experienced in the development of their capacity and capability for leadership. First, the included studies were read through until a sense of the whole was reached. Second, the extracted results, participant quotations [49] and paraphrases by the authors were aggregated. Third, in a collaboration among the three reviewers, these results were themed into subthemes and themes by similarity of meaning. The process continued until trustworthy themes were reached [39]. The themes were finally subjected to a meta-synthesis producing a single comprehensive set of synthesized results [46] and the effect size was calculated [49]. This process is illustrated in Table 4.

**Table 4:** Identified meta-synthesis, themes, subthemes and effect sizes

Meta-synthesis: HMMs develop capacity and capability through personal development processes empowered by context				
Study number	Theme 1: Personal development of capacity and capability		Theme 2: A need for contextual support	
	Effect Size: 96% (22 of 23 studies)		Effect Size: 91% (21 of 23 studies)	
	Subtheme 1a: A learning process Effect size: 96% (22 of 23 studies)	Subtheme 1b: Identification as a confident leader Effect size: 78% (18 of 23 studies)	Subtheme 2a: Networking Effect size: 83% (19 of 23 studies)	Subtheme 2b: Empowered by upper management Effect size: 65% (15 of 23 studies)
1	**+**	**+**		
2			**+**	**+**
3	**+**	**+**	**+**	**+**
4	**+**	**+**		
5	**+**	**+**	**+**	
6	**+**	**+**	**+**	**+**
7	**+**	**+**	**+**	**+**
8	**+**	**+**	**+**	**+**
9	**+**	**+**	**+**	**+**
10	**+**	**+**	**+**	
11	**+**	**+**	**+**	**+**
12	**+**	**+**	**+**	
13	**+**	**+**	**+**	
14	**+**		**+**	
15	**+**	**+**	**+**	**+**
16	**+**	**+**	**+**	**+**
17	**+**		**+**	**+**
18	**+**		**+**	**+**
19	**+**		**+**	**+**
20	**+**	**+**	**+**	**+**
21	**+**	**+**		**+**
22	**+**	**+**	**+**	
23	**+**	**+**		**+**

(+ indicates the number of studies in which a theme is addressed, while an empty spot indicates that a theme was not addressed)

## Results

The literature search of six databases identified 1853 studies. The search in the grey literature added 2025 studies. No relevant home pages were found [49, 59]. After duplicates were removed, the total number of studies was 3258. Screening by title and abstract excluded 3213 studies. The excluded studies did not meet the criteria of the PICo question used in this review: they did not involve HMMs or public healthcare, or they had quantitative designs. A total of 45 full-text articles were assessed for eligibility, and 26 were excluded. Of these articles, five had a different phenomenon of interest, three had no qualitative results, and 18 did not involve public healthcare. This inclusion process yielded 19 eligible studies. Through the included studies’ reference lists, we added four additional studies. Searching cited citations did not reveal further studies. This literature search ended in February 2019 with the inclusion of 23 studies.

The critical appraisal of methodological quality using the JBI-QARI instrument (Table 2) showed that only four [44, 66, 68, 75] of the 23 studies had positive answers to all ten of the questions assessed. Two of these studies were from Norway, one was from Canada, and one was from Australia. One of these studies was published in 2005, and the other three were published between 2015 and 2018. Two of the studies [74, 78] had only one positive answer to the ten questions assessed; these studies were from the USA and Sweden and were published in 2015 and 2016, respectively.

Question 6 concerned a statement locating the researcher culturally or theoretically. This question was addressed by 96% of the respondents. Ethical considerations, as part of questions 6–10, were not described in five of the studies [63, 69, 72, 74, 82], and an additional four studies [71, 73, 78, 80] were unclear in their descriptions. Question 7 *Is the influence of the researcher on the research, and* vice versa*, addressed,* had a very low achievement, 30%. Of the seven studies that addressed this concern, one was from Sweden, two were from Norway, one was from Australia, two were from Canada and one was from the USA/Taiwan; all of these studies were published between 2005 and 2018. Question 8, *Are participants, and their voices, adequately represented,* had a 43% score. Of the ten studies addressing this concern, four were from Australia, two were from Norway, two were from Canada, and one each was from Finland and the United Kingdom. These studies were published between 2005 and 2018.

In the context of the JBI-QARI, six studies [61, 62, 70, 73, 77, 80] were found to have methodological weaknesses. Of these studies, two were from Finland, two were from Canada and two were from Sweden, and they were published between 2005 and 2017. As stated by Sandelowski and Barroso [49, 59], qualitative research has no consensus on quality assessment or the use of quality criteria in systematic reviews. Methodological descriptions could also be affected by the editor and the context. The increased nuances in the data were considered to be of higher value than the disadvantages of inadequate methodological quality. Therefore, no studies were excluded for methodological reasons.

### Meta-summary of the extracted data

The studies were characterized by representing four continents. Nine studies came from North America [61, 64–66, 71, 73, 76, 78, 83], nine from Europe [47, 60, 62, 68, 69, 72, 77, 80, 82], four from Australia [63, 67, 70, 74], and one from Asia [79]. Eighteen of the 23 studies were published after 2009, and five were published between 2005 and 2007. Together, all of the studies included 482 participants. The participants were nurses in eighteen of the 23 studies, one study included physiotherapists, one included environmental services staff, one included midwives, one included physicians, and five of the studies did not describe the HMMs’ professional backgrounds.

The methods used were mainly individual interviews [60–62, 64, 65, 67, 68, 70–73, 75–80, 82] and focus groups [44, 61–63, 65, 66, 71, 74, 77, 79, 82], but field work [77, 79, 82], qualitative questionnaires [62, 79], workshops [67, 75], documentary reviews [67], essays [69], diagnostic assignments [70] and self-reflective diaries [79] were also employed. The analyses were mainly based on content analysis [66, 68, 70, 73, 74, 78–80], thematic analysis [61, 64, 69] and grounded theory [60, 72, 82], but an iterative approach [63], realist coding framework [67], critical hermeneutic analysis [44] and voice-relational method [75] were also used. One study was guided by interview questions and utilized NVivo [71], one used several interpretivist analyses [77], one described having used standard quantitative methodology [62], and two studies did not describe how data were analysed at all [65, 76].

The contexts of the studies included 20 studies in public hospitals of different levels and sizes, 15 studies in district hospitals (major health care facilities) [60, 62, 64, 65, 67–71, 73, 74, 77, 79, 82], twelve studies in general hospitals [44, 61, 62, 64, 67, 70, 73–76, 82] and one study in a specialized rehabilitation hospital [78]. Nine studies had a municipal healthcare context [44, 60, 63, 65–67, 72, 73, 82], including four studies in long-term care [44, 60, 63, 66], three studies in homecare [44, 66, 73], one study in a health centre [66] and one study focused on mental healthcare and public health [73].

### Meta-synthesis: HMMs develop capacity and capability through personal development processes empowered by context

**Fig. 2:**
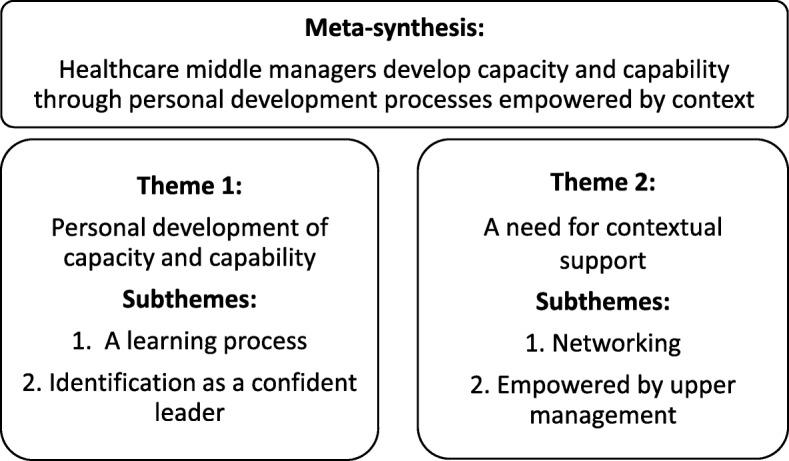
Conceptual model of the findings

The meta-synthesis *HMMs develop capacity and capability through personal development processes empowered by context* incorporated the results from 23 primary studies and was built on HMMs’ experiences of developing capacity and capability for leadership in a healthcare system characterized by high complexity. Two main themes were developed. The first main theme, *personal development of capacity and capability,* illustrated the development of capacity and capability through two subthemes: “a learning process” and “identification as a confident leader”. This main theme illustrated how HMMs experienced a personal drive for development on several levels with the purpose of maintaining leadership in a complex and changing context. The second main theme, *a need for contextual support,* was based on two subthemes: “networking” and “empowered by upper management”. This main theme illustrated how HMMs’ development processes were influenced by whether they experienced being in an empowering context, including by upper management and internal and external networks (see Fig. 2). The main themes had an effect size of 96 and 91%, respectively, and the subthemes were represented in no less than 65% of the studies (Table 4).

### Personal development of capacity and capability

Personal development of capacity and capability was experienced as a gradually changing process, adapting to a rapidly changing and complex context. This experience was described as a personal process that included acquiring the necessary competence involved in this process and finding oneself as a HMM, developing self-esteem, self-confidence and identity. This theme had two subthemes, a learning process and identification as a confident leader.

#### A learning process

The subtheme a learning process was present in 22 of the 23 studies when the development of capacity and capability was experienced as involving knowledge [44, 64, 65, 68–70, 77, 78, 82], reflection [44, 60, 62, 66, 68, 73], learning [44, 60, 68, 71, 77], self-knowledge [69, 82], concentration [62], passion, creativity [77], inspiration [65] and motivation [66]. This development was described as a learning process including coherence, flexibility, repetition, and short lectures [44]. The process was elaborated by one HMM:

*“Through reflections and discussions, I have become more conscious on my way of leading and how it can have consequences on employee health* [65]*”.*

The development of capacity and capability involved skills in engaging team members [71], promoting feedback processes and coaching [63], and developing skills in human resources [68, 69], leadership [72, 73], problem solving and decision making [64]. This development also involved skills in time management [62], project management [73], web-based learning and information technology [70]. HMMs experienced ineffective coping strategies [68, 80] and found that the development of effective coping strategies was useful [69]. Furthermore, the development of these skills involved proficiency in quality improvement, in the creation of a structured process to plan, lead and organize change [66, 67, 71–73, 80], in aligning aims [67, 77] and in achieving visible results [71]. It was also shown that HMMs developed positive prospects [62], progress [72] and the ability to balance challenges and opportunities [77]. The development of these skills was exemplified by one HMM:

*“I think that my leadership skills were there, however, they were developed further and helped me to increase the capability of what I was able to do and how I was able to grow as a leader* [73]*”.*

Several tools [65, 67, 73] were found to develop these skills, such as the Lean methodology [64], mental and conceptual models [72], learning tours [79], situational feedback, mindfulness exercises, an “I’m ok” diary [66] and clinical supervision [69]. The development of capacity and capability was experienced as providing broader perspectives [65, 69], understanding the bigger picture [71], and respecting human diversity [60]. The elements in these experiences of developing capacity and capability were contrasted by narratives from the participants’ typical work situations. As one HMM explained:

*“…in our work environment, especially in health care, we’re on a very strict deadline and there’s always a million and one things you need to complete in a day. And yes, production is one thing but if you don’t have time to reflect on your practices then you’re never going to change, you’re never going to improve the practice* [62]*”.*

HMMs considered access to continuous professional development important [80]. The results showed experiences of sink or swim [68, 75, 76], learning as you go [74, 82], and a personal need to seek management education [75].

#### Identification as a confident leader

The subtheme identification as a confident leader was present in 18 of the 23 studies when HMMs in the included studies experienced the development of capacity and capability as defining their personal leadership limits through establishing authority [60], changing attitudes, beliefs and knowledge [77, 78] about their role as a leader [69, 73, 78] and leadership [44, 67], and developing a leadership identity [60]. The start of this personal development process was described by one HMM as follows:

*“I didn’t know a lot of things nor the expectations of Nursing Unit Managers or ability required … You come into the role without knowledge and expectations of role* [64]*”.*

Entering the leader role, HMMs experienced a lack of self-confidence [44, 63, 64, 67, 68, 73]. Development occurred at the personal [60, 69, 70], managerial [60, 62], occupational [62, 82] and professional [79] levels and included confidence [44, 63, 64, 67, 68, 73], enhanced job performance and changes in leadership [64, 69, 70, 78], leadership styles [73] and leadership models [72], being a role model [62, 63, 77], gaining a voice [74], staff empowerment [63], accountability and commitment [67].

In 17 of the 23 studies [44, 61–65, 67–69, 72–74, 76, 78–80, 82] the purpose of the experienced development process was to contend with healthcare complexity. This development led to an increased intention to be a coach [63], less administrative, and more frontline, leadership [62], and dedicated time for and awareness of this complexity [72]. This result of the personal development process was described by one HMM as follows:

*“I don’t get very uptight about all those orders we get, instead I say yes, yes we’ve seen this before, now we’ll wait and see. So, the worst of it passes, because, like I usually say, what applies today doesn’t always apply tomorrow* [60]*”.*

### A need for contextual support

Although the development of capacity and capability was experienced as a personal process, the results showed that this process did not occur by itself. These results converge in the second main theme: a need for contextual support. This theme was experienced as a development of capacity and capability influenced by HMMs’ organizational and human contexts. This theme had two subthemes: networking and empowered by upper management.

#### Networking

The subtheme of networking was clearly present when HMMs described networks [44, 62, 64, 67, 77], workshops [44, 73] and multidisciplinary leader development courses [73, 82] as advancing their development, as well as when relational factors such as communication [63, 64, 68–70, 73], interaction [69, 70], reflective dialogue [65, 82], team work [71, 82], discussions [44, 73], conversations and storytelling [77], observing others [68, 71], group cohesiveness and new relationships [71] were brought forward. One HMM described the meaning of networking as follows:

*“The workshop has been very helpful from the networking side. You know there are Nurse Unit Managers all over the state with the same issues. You know you don’t think that you’re alone. Sometimes there, particularly out in the rural areas you feel like the problems that you’re facing are different from the problems that they’re facing in metropolitan areas or, you know, remote areas. But they’re not, a lot of them are much the same. So that’s been very helpful* [62]*”.*

A learning culture [61, 67] with support and encouragement from peer managers [65, 68, 75, 76], mentoring [68, 73, 74, 82], collaboration and sharing [64, 77], relational coordination [62, 66], feedback from staff [68, 82] and human resources [82] was experienced in the development of capacity and capability. Horizontal and vertical mentoring were valued [68]. Networks increased dialogue, cooperation and understanding [82], and knowledge sharing and were described as enhancing trusted interactions despite organizational and structural frames, providing a knowledgeable understanding of a complex context [44]. Informal networks were also found to aid in development [72].

The importance of networking was contrasted by narratives from the participants’ typical work situations, where HMMs described a feeling of loneliness [62, 66]. The development related to networks was experienced as important to be followed up at HMMs’ own workplaces [65]. The results showed some improved patient experiences [64] and limited impacts on managers’ behaviours or clinical practices [78]. The reason for this result was explained by one HMM:

*“Some Nursing Unit Managers haven’t been able to make changes because they simply haven’t had the time* [64]*”.*

#### Empowered by upper management

The subtheme empowered by upper management was presented by HMMs who experienced the need for resources [61, 67, 68, 70, 73], clear steering and vision, leadership structures [72, 82], plans [44, 78], information [61, 67], strategies [62, 82], communication [82], infrastructure [80] and rules [68]. A connection to the organization [77], maximized discretion [61], and a no-blame environment [77] were also among the results.

To develop capacity and capability, support [61, 65, 67, 68, 77, 80], trust [44, 63, 72, 77, 82], respect [60, 63, 72, 76, 77], feedback [68, 73, 82], influence [60], freedom [77] and participation [78] were experienced as central. The experiences of being empowered were described by one HMM:

*“We’ve had certain budget frameworks, of course, but besides that, we’ve been free to develop the organization the way we want to ourselves, as long as we’ve abided by the stipulated preconditions. And for that reason, I’ve been able to influence my job an awful lot* [60]*”.*

The need to be empowered by upper management was contrasted when HMMs experienced a lack of support [66, 68, 75, 76, 82] and feedback [66] from upper management and described that this had to be garnered [74]. HMMs experienced a need to be recognized, valued and empowered [62, 77] through autonomy [60, 67] and professional development [67]. One study described an experience of being empowered on the individual and interpersonal level but powerless on the system level [79]. The lack of support from upper management was explained by one HMM as follows:

*“I have to say that I have been through some crises here and I haven’t had support from anyone, no one in admin cared. I do try to deal with issues, but they’re no help, I’d hate to see anyone else go down the same path* [76]*”.*

## Discussion

This systematic review and meta-synthesis of 23 primary studies aimed to identify existing knowledge and critically discuss how HMMs experienced the development of the capacity and capability for leadership in a healthcare system characterized by high complexity. This meta-synthesis provided evidence of the development of capacity and capability based on a personal development process reinforced by an empowering context. In the following section, contrasts in the results are discussed from a critical hermeneutic perspective and in the context of the existing research. Finally, methodological considerations, strengths, limitations, and implications are discussed.

### Contrasts in the results of this meta-synthesis

The first main theme, *personal development of capacity and capability,* showed contrasts related to how HMMs described their need to develop a capacity and capability for leadership and how they experienced that their current complex organizational context in healthcare provided them the opportunity for such development. HMMs described their life world [40] as a feeling of being insecure and learning by doing, with a lack of leadership competence in approaching the position. Despite existing broad knowledge about the central role that competent HMMs have in healthcare [1–9], the results showed that it was left to chance and HMMs’ own initiative whether the necessary leadership skills were present or developed.

Although HMMs strove to develop their capacity and capability, the results did describe a personal development process. This meta-synthesis added new knowledge about the importance of building self-confidence as a HMM to develop capacity and capability. Reflection and interaction were experienced as important catalysts for these processes. In contrast, the results illustrated how HMMs experienced a life world [40] with a task-related typical work situation, which did not allow for time for reflection. HMMs experienced a lack of self-confidence in leadership, where upper management, as a part of the system world [40], had put them in a role they did not have the prerequisites to fulfil. These results suggest that although we have broad knowledge about healthcare as complex systems [20], this knowledge is not integrated in practice. This could be understood as examples of changeable dependent relationships that are taken for granted in the present healthcare system [41] and that are not to be questioned. Thus, healthcare remains guided and structured in traditional ways, despite the rapid changing and increasingly complex context [21, 22]. Consequently, the development of HMM’s capacity and capability will also be aimed towards the dominating task-oriented transactional leadership style and needs to be complemented with the capacities and capabilities of the more relational and transformative leadership perspectives [7, 8, 31, 34].

The second main theme, *a need for contextual support*, showed contrasts related to how HMMs described networks and to be empowered by upper management as essential to developing capacity and capability and how they experienced the lack of these in their present healthcare contexts. One study described how HMMs felt they needed to garner support [74], while another study described HMMs as powerless on the system level [79]. HMMs experienced support and feedback from their peer HMMs, but several studies described a lack of empowering support and feedback from upper management [66, 68, 75, 76, 82]. These results added to the existing knowledge describing a dominating top-down management in healthcare, HMMs’ loss of involvement and autonomy [3, 28], and the relevance of a change in leadership styles where transformative [7, 31] and relational leadership [8, 31] are argued to better relate to the present complex healthcare systems [7, 31]. Communicative rationality can only be accomplished through bottom-up social interaction, since the reality is known only to the participants of the processes [40]. Several of the included studies [44, 62, 64, 67, 77] described how HMMs experienced participation in different forms of networks as developing. Additionally, other relational aspects linked to interaction were emphasized as crucial. These issues stand out in contrast to HMMs’ life world experience of loneliness in their leadership role [62, 66] and added to the knowledge about complexity in interactions and complex systems based on dynamic networks [27].

These results show how healthcare are not recognized as unique and complex contexts, but instead are dominated by traditional management and organizational structures. The complexity in itself causes HMMs to take hold of their own development from the experience of not having the capacities and capabilities that are necessary, but they experience as though they stand alone in this process. In summary, the results elucidated a need to change the structures and approaches in the context of HMMs and in how HMMs are appointed and supported to ensure a strengthening development process in their leadership.

### Methodological strengths

The methodological strengths of this systematic review included a structured search of the literature and an examination of each primary study using the critical appraisal instrument JBI-QARI [46]. The a priori published, peer-reviewed protocol [12] and collaboration with two university librarians secured a well-prepared search and enhanced the study’s dependability and trustworthiness. The inclusion of sources from the grey literature extended the search base with studies not published in known databases, such as monographs, books, reports, guidelines or recently completed studies [49, 59]. Two different researchers, the first and third reviewers, conducted separate critical assessments of the primary studies and discussed the results until a common conclusion was reached. Despite noted methodological weaknesses, no studies were excluded. This approach protected against the loss of valuable data caused by primary studies’ shortcomings in the implementation and/or presentation of methodological choices. The critical appraisal showed that question 6, a statement culturally or theoretically locating the researcher, was addressed by 96%. This result is especially high and may represent a need to place the research and researcher, which is a recognized issue in qualitative research [92].

The included studies used different methods for qualitative data collection and analysis. This approach provided the review with an overall breadth and depth of knowledge, where different entrance points were used to arrive at the results. The included studies originated from several different contexts, nationalities and continents in developed Western countries and showed surprising homogeneity in the presented experiences of the participants. Thus, this evidence points to directions for approaching the future development of HMMs’ capacity and capability in both municipal healthcare and hospitals from an international perspective.

This systematic review benefited from the JBI Reviewer’s Manual [46] and Sandelowski and Barroso’s comprehensive framework for qualitative research synthesis [49]. The JBI revised model [46] clarified the conceptual integration of evidence generation, synthesis, transfer and implementation [48]. This model and manual added to the transparency of the review, as they provided a comprehensive guide to conducting and structuring the a priori published, peer-reviewed protocol [12]. The JBI-QARI [46] enhanced the dependability by providing methodological guidance on the critical assessment process. Sandelowski and Barroso’s framework helped advance the knowledge and develop the theory based on primary studies by aggregating target findings and offering valid guidelines for a meta-synthesis. Following the seven-step procedure added to the trustworthiness of the results by enhancing dependability [59]. Credibility was enhanced by quotations representing the participants in the primary studies and the collaboration among three different experienced researchers from different professions.

### Methodological limitations

The methodological limitations of this systematic review included that healthcare leadership and management are described by several and diverse concepts. The three-step search strategy following an a priori published, peer-reviewed protocol [12] defined and utilized an extensive range of them. However, we cannot exclude the possibility that using other search terms could have helped identify other contributions.

The search process included the identification of a larger number of articles (2025) from sources other than articles found in ordinary databases (1853). This approach could be seen as a sign of an inadequate search strategy, since a structured search would be expected to result in a larger number of findings. However, this is mainly the matter in the health sciences. This review presented healthcare leadership as a broad field of interest for different research traditions. As examples, Simpson [77] wrote in the field of adult education, and Tyan [79] wrote in the field of philosophical tradition. Additionally, the exclusion of 3213 studies after the screening of titles and abstracts could indicate a lack of search precision. However, this result is more likely a sign of a lack of a common language and keywords across disciplines. The sources of grey literature (Google Scholar, MedNar and ProQuest Dissertations and Theses Global) had fewer opportunities to limit the search [46]. These sources produced many irrelevant studies, which were excluded, but they also produced valuable studies not identified through other databases. Three of the included articles were a PhD thesis [79] and two master theses [68, 77] that were found only in ProQuest Dissertations and Theses Global.

This systematic review included studies in English, German or Nordic languages, which provides a possibility for publication bias. The exclusion of non-public healthcare led to the exclusion of most studies developed in the USA. This exclusion could indicate a loss of results. However, the differences in contexts were of such an extent that the limitation was valued as clarifying. Additionally, the exclusion of quantitative studies could mean that results were omitted. This exclusion was supported by the aim of this review: to identify and critically discuss HMMs’ experiences. The qualitative method was thus understood as expedient. Hewison [104] even suggested that the fragmented, reactive and interpersonal activity of management makes only qualitative research relevant.

The critical appraisal presented a low score in general, and only one question had a total score of 96%. However, this result may be due to guidelines from the journals and editors when publishing. Additionally, JBI-QARI was developed in a healthcare tradition, and the included studies were published in a variety of research traditions. In terms of effect size, 80% of the questions had over 61%. However, question 7, assessing researchers’ interference with research, and question 8, ethical assessment, negatively stand out with 43 and 30%, respectively. These questions are central to qualitative studies and could thus have been taken for granted and therefore not specified. However, this result could also mean that these important questions were neglected. One of the included studies [62] even referred to standard quantitative analysis methodology for qualitative analysis. Overall, the lack of arguments for the selection of methodology and self-reflection on the researcher’s influence contributes to the descriptions of Uhrenfeldt [43], who identified weaknesses in this area, even in qualitative research.

### Implications for healthcare and further research

Our study has important implications. This study provides evidence of the need for a changed approach in healthcare regarding both organizational structure and leadership methods, aiming to enable HMMs’ capacity and capability. The most important contribution this study provides is establishing connections between how HMMs develop capacity and capability by developing self-confidence in leadership through a learning process based on interaction in the complex system and an empowering approach from upper management. The facilitation of such development requires a change in how we organize and relate to management in healthcare. The change is needed to move from command and control to a leadership development process based on networking, interaction, trust and respect, clear structures and frameworks, support and feedback.

The context of the included studies was dominated by Western developed countries, especially from North America and Europe. This result may indicate that transferability to the context of developing countries requires further research. The contexts were mainly hospitals, which may be because hospitals are assumed to provide better feasibility for research, and it may also be an example of municipalities as a context in need of more health-related research. Although this PICo had a multidisciplinary approach to HMMs, the participants in the included studies were mainly nurses. This result may demonstrate that these positions are mainly held by nurses but could also show a need for further research on multi-disciplinary leadership at this level. The included studies did not provide results about whether or how HMMs’ development of capacity and capability changes practice or if this could be understood as solely a personal development process. Only one study showed some improved patient experiences [64]; another described how HMMs’ development of capacity and capability had a limited impact on managers’ behaviours and clinical practice [78]. Therefore, this systematic review did not provide evidence about whether HMMs’ development of capacity and capability reduced harm, improved patient safety, or strengthened the quality of healthcare. This question will be an important topic for future research.

## Conclusions

This meta-synthesis identified the established knowledge and critically discussed how HMMs experienced the development of their capacity and capability for leadership in a healthcare system characterized by high complexity as a personal process of building self-confidence, knowledge, skills and tools. The central role of HMMs in current healthcare organizations, structural constraining of leadership, the importance of a supportive top management, and how context influences leadership, have been demonstrated previously. However, this study added new evidence of how HMMs in public healthcare experience that the increasing complexity of healthcare changes which capacities and capabilities are necessary to develop, and how these skills must be developed by non-traditional methods. These methods are based on facilitating bottom-up development processes in an empowering context through interaction in networks and an empowering approach from upper management. This study also added new evidence about the importance of building self-confidence as a basis for leadership development processes. These results were in clear contrast to what HMMs described as their typical work situation, which was experienced as unprepared, lonely and with little support and feedback from upper management. The results showed that this field of research is dominated by nurse management; in this context, this study also adds new knowledge about HMMs with a multidisciplinary approach. In conclusion, this evidence is usable as a basis for politicians, administrators and healthcare managers to implement changes related to how we structure and lead international healthcare: a change in leadership development processes based on networking, interaction, trust and respect, clear structures and frameworks, support and feedback.

## Data Availability

Coding data from this qualitative review are available upon request from the corresponding author, TAH, at trude.a.hartviksen@nord.no.

## Abbreviations

HMMs: Healthcare Middle Managers
JBI: Joanna Briggs Institute
JBI-QARI: Qualitative Assessment and Review Instrument
JBI-SUMARI: System for the Unified Management, Assessment and Review of Information
MeSH: Medical Subject Headings
PICo: Participants, phenomena of Interest and Context
PRISMA: Preferred Reporting Items for Systematic Reviews and Meta-Analyses
